# SLEEP DISPARITIES AMONG ASIAN AMERICANS: IMPACTS OF DISCRIMINATION AND ACCULTURATIVE STRESS

**DOI:** 10.1093/geroni/igad104.0669

**Published:** 2023-12-21

**Authors:** Lee Sunmin

**Affiliations:** University of California Irvine, Irvine, California, United States

## Abstract

Although Asian Americans have shorter sleep duration and more sleep difficulty compared to Whites or Hispanics, research on Asians remains scarce. 60% of Asians are foreign-born and have high levels of limited English proficiency, which is positively associated with a range of immigrant stressors, such as discrimination and acculturative stress. Importantly, COVID-19 has exacerbated discrimination towards Asian Americans giving rise to ‘Asian hate’. In a study using data from the National Epidemiology Study of Alcohol and Related Conditions III (n=1,765), we found that individuals with high discrimination were sleeping 14.4 minutes less (SE: 6.0, p < .05) and had a higher prevalence of sleep difficulty (prevalence ratio (PR): 1.73, 95% Confidence Interval (CI): 1.33-2.24) compared to those with low discrimination. An interaction effect was observed in sleep difficulty by nativity. The association between discrimination and sleep difficulty was stronger among the U.S.-born than the foreign-born. In another study of Chinese and Korean Americans (n=400), Poisson models revealed that greater acculturative stress was associated with a higher prevalence of sleep disturbance (PR: 1.18, 95% CI: 1.06% to 1.31%). Interaction tests indicated that only women had a significant association between acculturative stress and sleep disturbance (PR: 1.30; 95% CI: 1.13 to 1.49), while the association was significant for individuals identifying as “very Asian” (PR: 1.21; 95% CI: 1.08 to 1.35), but not for those identifying as “mostly Asian” or “bicultural/western”. Future sleep health research in Asian Americans should consider the critical role of discrimination and acculturative stress.

